# A Hand-book on the Diseases of the Heart, and Their Homœopathetic Treatment

**Published:** 1882-09

**Authors:** 


### A HAND-BOOK ON THE DISEASES OF THE HEART, AND THEIR HOMŒOPATHIC TREATMENT. By W. P. Armstrong, M. D. Duncan Brothers: Chicago. 1882.

Dr. Armstrong first gives a short description of the anatomy and physiology of the heart; then follow chapters on diagnosis of heart diseases. After these, the various diseases are reviewed, and brief articles on their treatment given. The indications for the remedies are good, but brief, too brief.

As it is only in our *therapeutics* that we of the homœopathic school can compete with allopathic authors, our writers should pay more attention to this important department. At present they give entirely too much space to clinical history, pathology and the like. In these branches we cannot expect to rival the giants of the allopathic hospitals. Our hospital advantages are vastly inferior; our opportunities for clinical study so meagre. Let us, therefore, give more attention to therapeutics, and as this is cultivated more and more thoroughly, our success will be greater and greater, until finally it compels the allopaths to surrender their hospitals to us.

Dr. Armstrong's book is a good one, and we are glad to welcome it.

